# MEPE/OF45 as a new target for sensitizing human tumour cells to DNA damage inducers

**DOI:** 10.1038/sj.bjc.6605572

**Published:** 2010-02-09

**Authors:** P Zhang, H Wang, P S N Rowe, B Hu, Y Wang

**Affiliations:** 1 Department of Radiation Oncology, Thomas Jefferson University, Philadelphia, PA 19107, USA; 2 Department of Radiation Oncology of Winship Cancer Institute, Emory University School of Medicine, Atlanta, GA 30322, USA; 3 Department of Internal Medicine, Kidney Institute and Division of Nephrology, University of Kansas Medical Center, Kansas City, KS 66160, USA; 4 Department of Medical Molecular Biology, Beijing Institute of Biotechnology, Beijing 100850, China

**Keywords:** MEPE/OF45, CHK1, DNA damage

## Abstract

**Background:**

We recently identified matrix extracellular phosphoglycoprotein/osteoblast factor 45 (MEPE/OF45) as a new cofactor of CHK1 in rat cells. The aim of this study was to determine the role of human MEPE/OF45 (hMEPE/OF45 has ∼50% homology with rat MEPE/OF45 (rMEPE/OF45)) in affecting the sensitivity of human tumour cells to DNA damage.

**Methods:**

hMEPE/OF45 expression in different human tumour cell lines and its relevance to the resistance of cell lines to DNA damage inducers such as ionising radiation (IR) or camptothecin (CPT) were assessed. Cells lines stably expressing wild-type MEPE/OF45 or mutant MEPE/OF45 (with the CHK1 interactive key domain (amino acids 488–507) deleted) were established. Cell survival, G_2_ accumulation, CHK1 half-life and the CHK1 level in ligase 3 complexes were examined.

**Results:**

hMEPE/OF45 expression correlates with the resistance of cell lines to IR or CPT. Upregulating wild-type hMEPE/OF45 (but not mutant hMEPE/OF45) could stabilize CHK1 by reducing CHK1 interaction for its E3 ligases Cul1 or Cula4A; it increases the G_2_ checkpoint response and increases the resistance of tumour cells to IR or CPT treatment.

**Conclusion:**

hMEPE/OF45 could be a new target for sensitizing tumour cells to radiotherapy or chemotherapy.

Matrix extracellular phosphoglycoprotein/osteoblast factor 45 (MEPE/OF45) was originally cloned from a human oncogenetic hypophosphataemia tumour (Rowe *et al*, 2000) and then identified in rat bone-marrow-derived osteoblasts (Petersen *et al*, 2000). The murine homologue of MEPE/OF45 was reported the following year (Argiro *et al*, 2001). Since the identification of MEPE/OF45, its function related to bone metabolism has been widely investigated. We previously identified that MEPE/OF45, as a cofactor of CHK1, protects cells from DNA damage-induced killing in rat embryo fibroblast cells (Liu *et al*, 2009). However, because human MEPE/OF45 (hMEPE/OF45) has only ∼50% homology with rat MEPE/OF45 (rMEPE/OF45), it remained unclear whether hMEPE/OF45 has similar function to rMEPE/OF45.

CHK1 is one of the essential checkpoint proteins involved in cellular response to multiple DNA damage inducers (Liu *et al*, 2000; Zhao *et al*, 2002). It is believed that upregulated CHK1 protects cells from DNA damage-induced cell killing, including that induced by ionising radiation (IR) and camptothecin (CPT) (Hu *et al*, 2001, 2005a; Zhao *et al*, 2002; Wang *et al*, 2002b; Brown and Baltimore, 2003), by promoting homologous recombination repair (HRR) (Sorensen *et al*, 2005; Hu *et al*, 2005b). Despite the importance of CHK1 in DNA damage response, the regulation of CHK1 in mammalian cells is not well understood, partly because of its essential nature (Liu *et al*, 2000; Lam *et al*, 2004).

In this study, on the basis of our previous report about the new role of rMEPE/OF45 in protecting cells from DNA damage-induced killing (Liu *et al*, 2009), we investigate whether hMEPE/OF45 is generally expressed in different types of tumour cells, whether the expression levels were relevant to the resistance of tumour cells to DNA damage inducers and whether, similar to rMEPE/OF45, hMEPE/OF45 (as a cofactor of CHK1) could be a new target for sensitizing human tumour cells to DNA damage inducers.

## Materials and methods

### Cell lines and antibodies

All human tumour cell lines used in this study were derived from ATCC. These cell lines were grown in DMEM medium supplemented with 10% calf serum. The antibody against hMEPE/OF45 was as described (Rowe *et al*, 2000, 2004). Purified HA-tagged JNK2 protein (sc-4062) and antibodies against CHK1 (sc-8408 and sc-7898), HA (sc-805, sc-7392), PCNA (sc-56), *α*-Tubulin (sc-8035), Cul4 (sc-10782) and ubquitin (Ub) (sc-9133) were purchased from Santa Cruz Biotechnology Inc (Santa Cruz, CA, USA). The antibody against Cul1 was purchased from Zymed Laboratories (San Francisco, CA, USA) and the antibody against H2A (07-146) was purchased from Upstate Biotechnology (Lake Placid, NJ, USA).

### Plasmid construction and transfection

The full-length cDNA of wild-type *hMEPE/OF45* or mutant *hMEPE/OF45* (with the sequence encoding 488–507 amino acids (aa) deleted) was ligated with HA tag at the C terminus by PCR. *hMEPE/OF45* was then cloned into pLenti6/V5-D-TOPO vector (Invitrogen), according to the manufacturer's protocol. The HA tag was inserted into the plasmid. The primers used for wild-type hMEPE/OF45 are: forward, 5′-CACCATGGGAGTTTTCTGTGTGGGACTACTCC TTTTCAGTGTGACCTG-3′ and reverse, 5′-AGGTCCTCCCAGGCTGGCATAGTCAGGCAC GTCATAAGGATAGTCACCATCGCTCTCACTTGAAC-3′. The procedure to construct mutant hMEPE/OF45 has been described in Supplementary Figure S1. The primers for fragment 1 (1–477 aa) are F1, ACGGATCCATGCGAGTTTTCTGTGTGGGACTACTCC and R1, TACTGTCATCCTTATTCCGTGTAGA, and for fragment 2 (507–514 aa) are F2, ACGGAATAAGGATGACAGTAGTGAGTCATC and R2, AAGAATTCGTCACCATCGCTCTCACTTG. For lentivirus production, pLenti6/V5-D-TOPO was co-transfected with packaging vectors into human 293 cells and the supernatant was harvested after 48 h. The virus was filtered through a Millex-HV PVDF filter (hole diameter: 0.45 *μ*M). Titres were determined by infecting 293 cells with a serial dilution of the concentrated virus. For a typical preparation, the titre was approximately 2- to 7 × 10^4^ TU ml^−1^. For infection of 293 cells, 30–50% confluent cells were incubated with a suitable amount of virus particles and 10 *μ*g ml^−1^ polybrene for 8–16 h at 37°C. A volume of 10 *μ*g ml^−1^ of blasticidin (Invitrogen) was used to select stably expressed cells.

### Immunoprecipitation

Immunoprecipitation was performed as described previously (Wang *et al*, 2002a). The samples were washed with buffer (0.5% NP-40, 1 mM Na_3_VO_4_, 5 mM NaF and 0.2 mM PMSF in PBS buffer) and boiled in 30 *μ*l of protein loading buffer. They were loaded onto 10% Tris-PAGE gel, followed by the standard western blot.

### Cell sensitivity to DNA damage inducers and flow cytometry measurement

Cell sensitivity to IR or to CPT was determined by clonogenic assay as described previously (Hu *et al*, 2001; Wang *et al*, 2002b). The G_2_ accumulation of cells after radiation was detected by flow cytometry as described previously (Hu *et al*, 2001). Briefly, 2 × 10^5^ cells were plated in 60 mm dishes, with 3 ml of growth medium. After 30 h, the cells were exposed to 6 Gy and returned to 37°C. At different times thereafter, the cells were trypsinised and fixed in 70% ethanol. Cells were stained in solution (62 *μ*g ml^−1^ RNase A, 40 *μ*g ml^−1^ propidium iodide and 0.1% Triton X-100 in PBS buffer) at room temperature for 1 h. The distribution of cells in the cell cycle was measured using a flow cytometer (Coulter Epics Elite, Beckman Coulter Inc., Atlanta, GA, USA).

### Statistical analysis

Differences between treatment groups were analysed using statistical software (*t*-test). *P*-values <0.05 were regarded as significant.

## Results and discussion

### MEPE/OF45 presents in all kinds of human dividable cell lines tested in our laboratory

**Figure 1:**
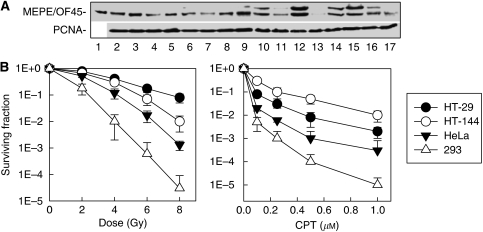
MEPE/OF45 expressed in dividable human cells. (**A**) Whole-cell lyses were prepared. The antibody against hMEPE/OF45 was as described (Rowe *et al*, 2000, 2004). Data shown are the images of western blot analysis. Expression of MEPE/OF45 in human normal and tumour cell lines. 1, purified MEPE/OF45 protein (Rowe *et al*, 2000); 2, MRC5 (primary human fibroblast); 3, MRC5-SV1 (transformed MRC5); 4, C3-ABR (transformed human lymphoblasts); 5, HeLa (human cervical cancer); 6, HCC1937 (breast cancer); 7, U87MG glioblastoma; 8, SAOS2 (osteosarcoma); 9, HT-29 (colorectal cancer); 10, A549 (lung carcinoma); 11, PC-3 (prostate cancer); 12, HT-144 (melanoma); 13, 293 (kidney cancer); 14, SQ20B (laryngeal cancer); 15, Capan-1 (pancreatic cancer); 16, OVCAR10 (ovarian adenocarcinoma); 17, MCF-7 (breast cancer). (**B**) Sensitivities of different human cell lines to DNA damage inducers. The sensitivities of cell lines to various doses of IR or CPT exposure (24 h) were examined using clonogenic assay. Bars show the mean±s.e. of the percentage of colonies from non-treated control cells. Data were obtained from three independent experiments.

We previously identified that rMEPE/OF45 could protect rat cells from DNA damage-induced killing (Liu *et al*, 2009). However, because the amino-acid sequence of hMEPE/OF45 (525 amino acid) has ∼50% homology compared with that of rMEPE/OF45 (435 amino acid) (Argiro *et al*, 2001), it remained unclear whether hMEPE/OF45 has a function similar to rMEPE/OF45. To investigate whether MEPE/OF45 was important for human cells to respond to DNA damage, we examined the MEPE/OF45 expression level in different human cell lines, including primary fibroblast cells, transformed fibroblast cells, transformed lymphoblast cells and tumour cell lines from different human tissues. These cell lines all showed a positive expression of MEPE/OF45, although the levels of expression varied (Figure 1A), indicating that MEPE/OF45 expresses in replicating cells. The signals of human MEPE/OF45 showed two bands (Figure 1A), which might be due to alternatively spliced *MEPE/OF45s*. Our group recently identified a new exon of *hMEPE/OF45* between formerly identified exons 3 and 4 (GenBank accession no. DQ854717). The functions of the different forms of MEPE/OF45 need to be investigated further. Interestingly, the levels of MEPE/OF45 in different human tumour cell lines broadly correlate with the resistance of cell lines to DNA damage inducers (Figure 1B). The higher the level of MEPE/OF45 was in the cell line, the more resistant the cell line was to IR or CPT. There were 293 cells with the lowest level of MEPE/OF45, and they were most sensitive to IR or CPT. In addition, we reported that knocking down MEPE/OF45 could sensitise HeLa cells to DNA damage inducers (Liu *et al*, 2009). These results indicate that MEPE/OF45 protects human cells from DNA damage-induced killing.

### Human MEPE/OF45 interacts with CHK1 and stabilises CHK1

**Figure 2:**
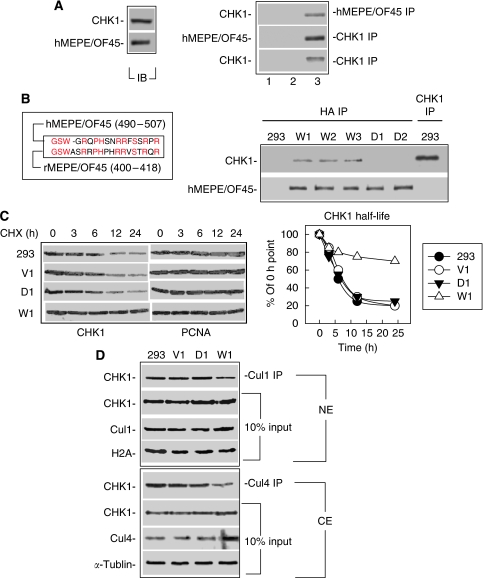
MEPE/OF45 interacts with CHK1 and stabilises CHK1. (**A**) Whole-cell lyses were prepared from HeLa cells. Signals were detected from immunoprecipitation (IP) results. Lanes 1 and 2 show the results from IP experiments that used general mouse or rabbit serum as the negative controls. Lane 3 indicates the western blot signals of CHK1 or of MEPE/OF45 from MEPE/OF45 or CHK1 antibody–immunoprecipitated complex. (**B**) Left: the corresponding amino-acid sequence of rMEPE/OF45 (400–418) in hMEPE/OF45 is 490–507. Right: MEPE/OF45 interacts with CHK1 in human 293 cell lines. Western blot (IB) with CHK1 or with MEPE/OF45 antibody was used to detect the signals of MEPE/OF45 interacting with CHK1 from CHK1 or HA immunoprecipitated (IP) samples. V1: 293 cells transfected with vector alone; W1–W3: 293 cells transfected with wild-type hMEPE/OF45; D1–D2: 293 cells transfected with mutant hMEPE/OF45 (amino acid 490–507) deleted. (**C**) CHK1 half-life in different cell lines: cells were treated with 100 *μ*g ml^−1^ CHX at different times. Whole-cell lyses were prepared. CHK1 signals were detected by western blot. The CHK1 levels were plotted using PhosphorImager (GE Healthcare Bioscience Corp., Piscataway, NJ, USA) with software (ImageQuant). (**D**) MEPE/OF45 affects CHK1 interacting with E3 ligases in human cells. Extracts were prepared from these cells using an NE-PER kit (Pierce, Rockford, IL, USA). The extracts (500 *μ*g of cytoplasmic extracts or 300 *μ*g of nucleic extracts) were mixed with protein A that was conjugated with Cul1 or Cul4 antibody for IP. CHK1 antibody was used for immunoblotting. H2A or *α*-tubulin was used as an indicator for nuclear or cytoplasmic extracts.

We previously reported that rMEPE/OF45 protected cells from DNA damage-induced killing through its interaction with CHK1 (Liu *et al*, 2009). We wanted to determine whether similar to rMEPE/OF45, hMEPE/OF45 could also interact with CHK1. To study the relationship of hMEPE/OF45 with CHK1, we first synchronised HeLa cells and examined the expression of MEPE/OF45 at different phases of the cell cycle, as CHK1 expression depends on the cell cycle (Kaneko *et al*, 1999). The results showed that similar to CHK1, MEPE/OF45 expressed low in the G_1_ phase and high in the S and G_2_ phases (Supplementary Figure S2), suggesting a correlation between these two proteins. We further examined the interaction between hMEPE/OF45 and CHK1 using an immunoprecipitation (IP) approach in HeLa cells. The results showed that, similar to rMEPE/OF45, hMEPE/OF45 also interacted with CHK1 (Figure 2A). The key domain of rMEPE/OF45 was 400–418 amino acids (Liu *et al*, 2009). The corresponding amino-acid sequence in hMEPE/OF45 is 490–507 (Argiro *et al*, 2001; Figure 2B, left). To study whether CHK1 was stabilised when interacting with hMEPE/OF45, as it was when interacting with rMEPE/OF45, we generated the constructs encoding HA-tagged either wild-type hMEPE/OF45 or mutant hMEPE/OF45 with amino acid 488–507 deleted (Supplementary Figure S1). We expressed the wild-type hMEPE/OF45 or mutant hMEPE/OF45 in 293 cells, as this cell line had the lowest level of MEPE/OF45 in the human tumour cell lines tested in our laboratory (Figure 1A). The results showed that wild-type hMEPE/OF45 but not mutant hMEPE/OF45 could interact with CHK1 (Figure 2B, right), indicating that the 488–507 amino acids are the key domain for hMEPE/OF45 to interact with CHK1.

We were then interested in determining whether similar to the key domain of rMEPE/OF45, the key domain of hMEPE/OF45 also had an essential role in stabilising CHK1 by reducing the interaction of CHK1 for its E3 ligases, Cul1 or Cul4A. For this, we first compared the half-life of CHK1 between wild-type hMEPE/OF45-expressing cells and mutant hMEPE/OF45-expressing cells.

The results showed that the half-life of CHK1 in the wild-type hMEPE/OF45-expressing cells (W1) is longer than that in parental cells (293), the 293 cells transfected with the vector alone (V1) and the 293 cells transfected with mutant hMEPE/OF45 (D1) (Figure 2C). These results indicate that the interaction domain is essential for hMEPE/OF45 to stabilise CHK1. We then compared the CHK1 levels in its E3 ligase complexes with wild-type hMEPE/OF45-expressing cells and mutant hMEPE/OF45-expressing cells. Results showed that although the total levels of CHK1 in either cytoplasmic extracts (CE) or nuclear extracts (NE) are similar to all types of cells (293, V1, D1 and W1), the CHK1 levels in Cul1 or Cul4A IP complexes in W1 cells were less than that in 293, V1 or D1 cells (Figure 2D). Additional data showed that when hMEPE/OF45 was knocked down, ubiquitinated CHK1 was increased (Supplementary Figure S3A and B). These results indicate that although hMEPE/OF45 has ∼50% homology with rMEPE/OF45, its C-terminal amino-acid sequence, including the key domain to interact with CHK1, is relatively conserved, and similarly functions to stabilise CHK1.

### Wild-type but not mutant hMEPE/OF45 renders tumour cells resistant to DNA damage inducers

**Figure 3:**
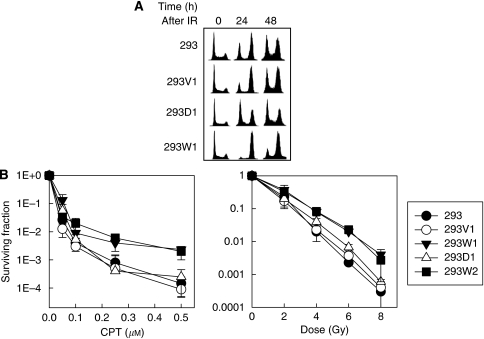
Upregulating wild-type MEPE/OF45 but not mutant MEPE/OF45 deleted the key domain that interacted with CHK1, making the tumour cells resistant to DNA damage inducers. (**A**) Comparison of the G_2_ accumulation of 293 cell lines (293, 293V1, 293W1 and 293D1) at different times after IR (6 Gy). The *P*-values between 293W (including 1 and 2) and other groups are <0.01. (**B**) Comparison of the sensitivities of 293, 293V1, 293W1, 293D1 and 293W2 cells to IR or CPT treatment (16 h). Data represented the average of three independent experiments and were expressed as the percentage of cell colonies without treatment. Data were obtained from three independent experiments.

To further examine whether hMEPE/OF45 was similar to rMEPE/OF45, and could protect cells from DNA damage-induced killing through interaction with CHK1, we compared the effects of wild-type hMEPE/OF45 and mutant hMEPE/OF45 on the G_2_ checkpoint response and the sensitivity of 293 cells to DNA damage inducers. The results showed that wild-type hMEPE/OF45 could increase the G_2_/M accumulation of cells after IR, but not mutant hMEPE/OF45 (Figure 3A). Similarly, knocking down hMEPE/OF45 reduced IR-induced G_2_ accumulation (Supplementary Figure S3A and C). These results further support that hMEPE/OF45 functions through the CHK1 pathway to affect cell response to DNA damage, because this kind of checkpoint response, G_2_-phase cell accumulation, is ATM independent (Xu *et al*, 2002) but CHK1 dependent (Wang *et al*, 2003). More importantly, the results showed that wild-type hMEPE/OF45 could increase resistance of the cells to either IR or CPT treatment, but not mutant hMEPE/OF45 (Figure 3B). The *P*-values between 293W (including 1 and 2) and other groups are <0.01. These results indicate that hMEPE/OF45 protects cells from DNA damage-induced killing depending on its interaction with CHK1. It is known that CHK1 promotes HRR through regulation of checkpoint activation (Sorensen *et al*, 2005; Hu *et al*, 2005b). We believe that hMEPE/OF45 increases the CHK1 level by stabilising CHK1, which promotes HRR and protects cells from DNA damage-induced killing.

### Conclusion

Taken together, the results of our study indicate that MEPE/OF45 protecting human cells from IR- or CPT-induced killing mainly depends on its interaction with CHK1. The specific molecular mechanism for this protective role of MEPE/OF45 could be linked to MEPE/OF45 stabilising CHK1 by reducing CHK1 degradation. Because *CHK1* is an essential gene (Liu *et al*, 2000; Lam *et al*, 2004) and MEPE/OF45 is not (Gowen *et al*, 2003), our study results indicate that MEPE/OF45 is a new cofactor of CHK1, which could be a new target with less side effects for sensitizing tumour cells to DNA damage inducers and could benefit cancer treatment.
