# Supplementary material for: MEPE/OF45 as a new target for sensitizing human tumour cells to DNA damage inducers
Source: Br J Cancer. 2010 Feb 9;102(5):862–6. doi: 10.1038/sj.bjc.6605572 (PMC2833259; doi:10.1038/sj.bjc.6605572)
Supplement: Supplementary Figures S1–S3 [file 6605572x1.pdf]

Supplementary information

MEPE/OF45 as a new target for sensitizing tumor cells to DNA damage inducers

Piyan Zhang, Hongyan Wang, Peter S.N. Rowe, Baocheng Hu and Ya Wang

Supplementary Figure Legends:

Figure S1 Description for designing construct of mutant hMEPE/OF45: To delete 488-507 aa (red color) from hMEPE/OF45, PCR was used to amplify fragment 1 with primers KoF1 and KoR1 and fragment 2 with primers KoF2 and KoR2. The mixed fragment 1 and fragment 2 were used as the template and the mutant hMEPE/OF45 was amplified by using PCR with primers KoF1 and KoR2. Primers KoR1 and KoF2 have 20 complementary matched base pairs (yellow and blue). The pink color represents the digest sites for subclone.

Figure S2 MEPE/OF45 expression associates with CHK1 in different cell cycles. G1 phase cells: 100% confluent HeLa cells were subjected to serum starvation (medium with 0.5% fetal bovine serum) for 72 h followed by 1 h of release (medium with 10% fetal bovine serum). S and G2/M phases: HeLa cells were synchronized by using double thymidine block. 25% confluent HeLa cells were cultured in the presence of 2 mM thymidine for 19 h, and released into fresh medium for 9 h. They were then incubated in the presence of 2 mM thymidine for 16 h before released into fresh medium. S phase cells were collected 30 min after the release and G2/M phase cells were collected 4.5 h after the release. NS: non-synchronized cells; G1: cells synchronized in G1 phase; S: cells were synchronized in S phase; G2/M: cells were synchronized in G2/M phases. Top panel: cells in different cell cycles were measured by flow cytometry. Bottom panel: the

levels of CHK1 and MEPE/OF45 were measured by Western Blot. PCNA acts as the internal loading controls.

Figure S3 MEPE/OF45 affects DNA damage-induced checkpoint response. (**A**) Effects of *MEPE/OF45* siRNA on the target protein. Human *MEPE/OF45 (hMEPE/OF45)* siRNA (5'-AAC TAA GCA AAG CTG TGT GGA-3') was designed to specifically target the sequence of 74-95 from the start codon region of the *hMEPE/OF45* cDNA. These siRNAs were synthesized by Dharmacon, Inc. Scrambled duplex RNAs (Dharmacon, Inc.) were used as the control transfection. The RNAs were delivered to the cells by OLIGOFECTAMINE™ (Invitrogen Corp.), according to the manufacturer's instructions. The cells were analyzed at 36-48 h post-transfection. (**B**) MEPE/OF45 protects CHK1 from DNA damage-stimulated ubiquitination. Left panel: Ubiquitine (Ub) attached to CHK1 at different times after the cells were exposed to 20 Gy. Whole cell extracts were prepared. Ub attached to CHK1 was detected at ~60 kD position from CHK1 immunoprecipitated (IP) complex. Coomassie brilliant blue-stained IgG 25 kD bands act as internal loading controls. The bars showed the mean (from two independent experiments) of the percentage of the level of ubiquitinated CHK1 at 0 h point. Right panel: the effect of *hMEPE/OF45* siRNA on the level of ubiquitinated CHK1 following IR (20 Gy). The bars showed the mean $\pm$ s.d. from three independent experiments. (**C**) G2 arrest of HeLa cells that were treated with control RNA or *hMEPE/OF45* siRNA was measured by flow cytometry at different times after the cells were exposured to IR (2 Gy).

KoF1	ACGGATCC ATGCGAGTTTTCTGTGTGGGACTACTCC	*hMEPE/OF45* 1-1461 bp
KoR1	TACTGTCATC CTTATTCCGTGTAGA	
KoF2	ACGGAATAAG GATGACAGTAGTGAGTCATC	*hMEPE/OF45* 1522-1575 bp
KoR2	AAGAATTC GTCACCATCGCTCTCACTTG	

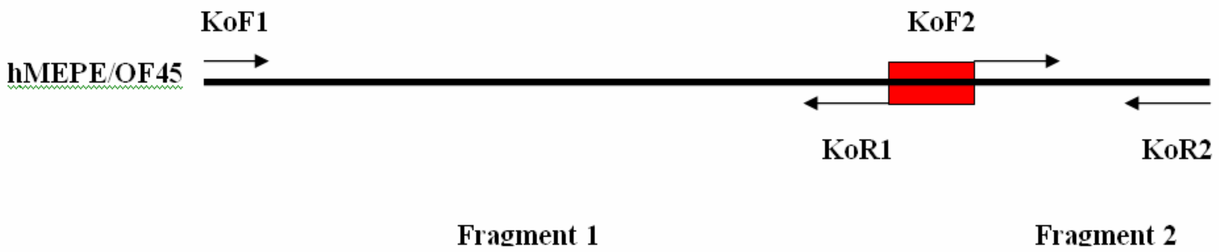

Figure S1

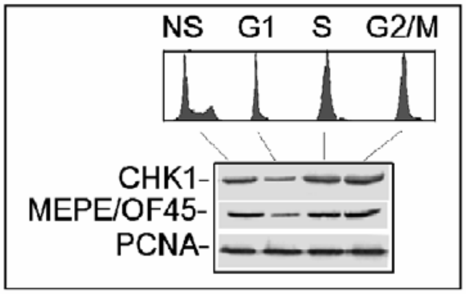

Figure S2

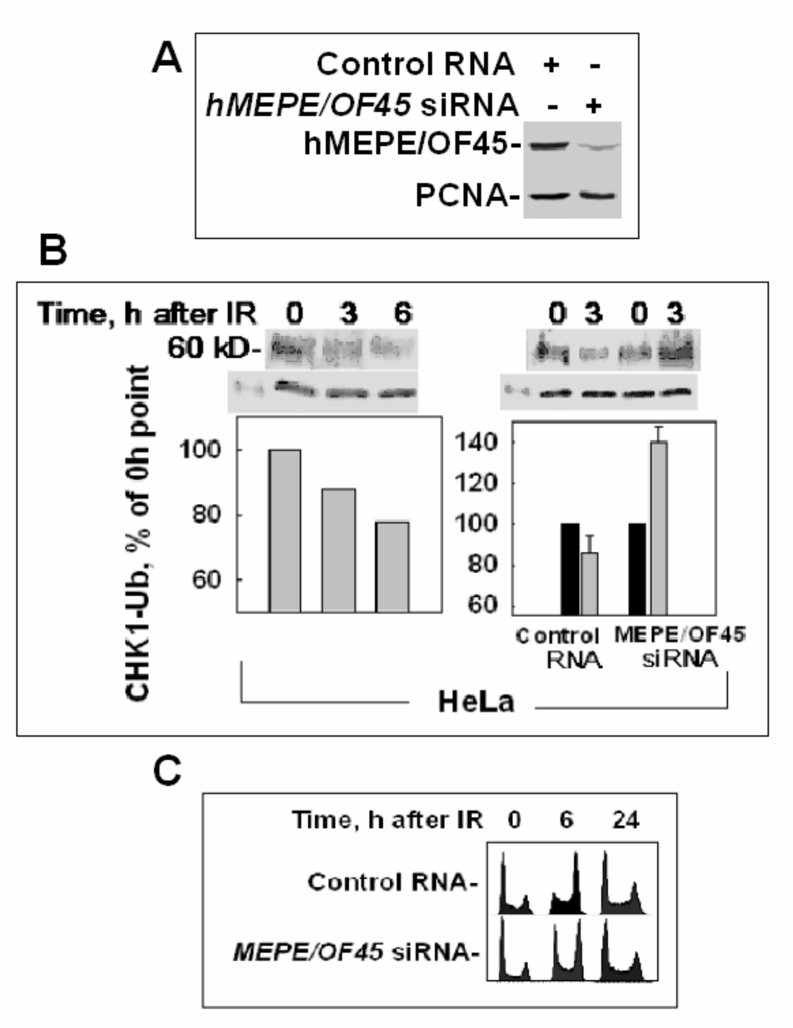
A
Control RNA + -
hMEPE/OF45 siRNA - +
hMEPE/OF45-
PCNA-
B
Time, h after IR 0 3 6
0 3 0 3
60 kD-
CHK1-Ub, % of 0h point
100
80
60
140
120
100
80
60
Control RNA
MEPE/OF45 siRNA
HeLa
C
Time, h after IR 0 6 24
Control RNA-
MEPE/OF45 siRNA-

Figure S3
